# Mood States, Physical Activity, and Sleep Quality in University Students: A Cross-Sectional Study in Educational Settings

**DOI:** 10.3390/healthcare14183083

**Published:** 2026-09-19

**Authors:** Laura García-Pérez, Paula Martínez Izaguirre, Gema Torres-Luque, Rosario Padial-Ruz

**Affiliations:** 1 Department of Didactics of Musical, Visual Arts, and Corporal Expression, Faculty of Humanities and Education Sciences, University of Jaén, 23071 Jaén, Spain; lgperez@ugr.es; 2 Department of Didactics of Musical, Visual Arts, and Corporal Expression, Faculty of Education, University of Granada, 18071 Granada, Spain; paulamartinz@correo.ugr.es (P.M.I.); rpadial@ugr.es (R.P.-R.)

**Keywords:** mood states, physical activity, sleep quality, sleep duration, university students, psychological well-being, health promotion, higher education, psychosocial factors, integrated care

## Abstract

**Highlights:**

**What are the main findings?**

- Poor sleep quality is highly prevalent among university students and is associated with higher scores in depression, fatigue, anger, and tension, whereas good sleep quality is associated with higher vigour.
- Higher PA levels are associated with greater vigour, whereas inactive/low-PA students generally show a less favourable mood-state profile.

**What are the implications of the main findings?**

- Health promotion strategies in higher education should prioritise sleep quality, not only sleep duration, as a key component of students’ affective well-being.
- Universities should promote accessible PA programmes together with sleep-health and psychosocial support strategies as part of integrated health promotion.

**Abstract:**

**Background/Objectives:** University students face academic, social, and lifestyle-related demands with the potential to influence affective functioning. Mood states provide information on everyday emotional profiles and may be associated with physical activity (PA) and sleep. This study analysed mood states and examined their relationship with physical activity, sleep quality, and sleep duration, considering sociodemographic and academic variables. **Methods:** A cross-sectional study was conducted with 1469 university students from Granada, Spain. Mood states were assessed using the Profile of Mood States, physical activity with the International Physical Activity Questionnaire–Short Form, and sleep quality and duration with the Pittsburgh Sleep Quality Index. Mann–Whitney U tests, Kruskal–Wallis H tests, and Spearman’s *rho* correlations were performed. **Results:** Poor sleep quality was observed in 81.4% of participants, while mean sleep duration was 6.96 ± 1.18 h/night. Students with poor sleep quality showed higher scores in depression, fatigue, anger, and tension than those with good sleep quality (all *p* < 0.001), whereas students with good sleep quality showed higher vigour (13.79 ± 5.39 vs. 11.59 ± 5.36; *p* < 0.001). Inactive/low-PA students showed higher depression and fatigue scores than more active groups, whereas the high-PA group showed the highest vigour scores. **Conclusions:** Mood-state profiles were associated with sleep quality, PA, and psychosocial and academic factors. Health promotion strategies in higher education should prioritise sleep quality, accessible PA, recovery, time management, and support for vulnerable profiles.

## 1. Introduction

The university stage represents a transitional period between late adolescence and early adulthood, characterized by new academic demands, changes in lifestyle habits, and personal and social adjustments that may affect students’ well-being [1]. Previous literature has shown that entry into and adaptation to university often coincide with a particularly sensitive developmental phase, in which students must cope with academic pressure, increased responsibilities, and simultaneous personal, family, social, and academic demands [2,3]. In this context, evidence from systematic reviews has reported that a considerable proportion of university students present high levels of anxiety and other indicators of psychological distress, reinforcing the need to study affective variables that are closer to students’ everyday experiences, such as mood states [4,5].

Unlike emotions, mood states are usually understood as more sustained, diffuse, and subjective affective processes, which are not always linked to a specific triggering stimulus. From a functional perspective, they constitute a relevant component of psychological adjustment, as they influence perceived well-being, disposition toward activity, self-regulation, and cognitive processes related to academic performance. Within this framework, the Profile of Mood States (POMS) has become a widely used instrument for the multidimensional assessment of affective states. Its structure allows the evaluation of negative dimensions, including tension, depression, anger, and fatigue, together with a positive dimension, vigour. In the Spanish population, the POMS has shown adequate psychometric properties, confirming its validity for analysing affective profiles in this study [6].

Recent literature suggests that a more positive affective disposition is associated with higher levels of life satisfaction, resilience, and psychological well-being, variables that may significantly influence cognitive functioning and students’ academic adjustment [7]. In contrast, negative affective states such as anxiety, stress, anger, or fatigue may interfere with selective attention, reduce memorisation capacity, and hinder information retention [8,9]. Conversely, positive affective states, such as vigour, have been associated with greater cognitive flexibility [10]. Emotional well-being may also influence students’ academic trajectories, since lower emotional well-being has been associated with poorer academic performance and higher university dropout rates [11]. Moreover, under academic stress, some students may adopt maladaptive coping strategies, such as alcohol or tobacco use, which have been associated with poorer physical health and increased negative mood states at the end of the academic year [12].

In this regard, it is important to consider modifiable lifestyle factors that may influence university students’ affective profiles. Among these factors, physical activity (PA), sleep quality, and sleep duration have shown consistent associations with emotional regulation, perceived well-being, and affective functioning [13,14,15]. These behaviours are particularly relevant from an integrated health promotion perspective, as they connect physical, psychological, and psychosocial dimensions of health and may be addressed through preventive and educational strategies.

Sleep is an essential biological process closely linked to emotional regulation. Reference institutions, such as the National Institute of Neurological Disorders and Stroke, highlight that sleep is a vital component of daily life and a determinant of brain functioning, influencing processes such as the clearance of brain metabolites and memory consolidation [16]. Consequently, sleep restriction or poor sleep quality, particularly when sustained over time, may contribute to poorer psychological well-being and less adaptive affective regulation [17]. In this line, the concept of “sleep health” introduced by Buysse, proposes a multidimensional view of rest that includes duration, regularity, continuity, subjective satisfaction, and daytime sleepiness, all of which are closely related to physical and psychological well-being [18]. Among university students, sleep disturbances are frequent, and several studies have shown that a considerable proportion of students experience sleep problems associated with poorer physical and psychological status, lower academic performance, and reduced quality of life [19]. In addition, negative affective states such as anxiety, fatigue, or anger may disrupt normal sleep rhythms, making it more difficult to initiate and maintain nocturnal rest [20]. Conversely, positive psychological variables, such as vigour and mindfulness, appear to have a protective role in sleep quality by promoting more adaptive affective management [20,21]. From a biological perspective, the associations between sleep, physical activity, and affective functioning may involve neuroendocrine regulation, inflammatory processes, and circadian mechanisms. These pathways may contribute to emotional regulation and psychological functioning; however, they were not directly assessed in the present study.

PA is also an essential component of overall health and is defined as any bodily movement produced by skeletal muscles that requires energy expenditure [22]. Available evidence suggests that PA may contribute to improved mood states through physiological and psychological mechanisms, including modulation of the inflammatory response, cortisol regulation, and improvements in perceived self-efficacy and well-being. Conversely, unhealthy lifestyle habits, such as insufficient regular PA, may affect bodily systems and be reflected in more unfavourable mood profiles, characterized by greater confusion, fatigue, and lower vitality [23]. In this context, it is particularly relevant that a recent study reported that 32.8% of university students did not meet the PA recommendations established by the World Health Organization, reinforcing the importance of analysing this behaviour as a determinant of affective functioning [24]. Furthermore, in the university context, PA has been shown to be a useful strategy for improving mood profiles by reducing tension, anger, and fatigue, and by increasing vigour and personal satisfaction.

Although previous studies have examined well-being in relation to factors such as PA or sleep, these lifestyle behaviours have often been analysed independently. In the university context, there is still limited evidence examining the relationship between these variables and the multidimensional profile of mood states, as most studies tend to focus on broader indicators of mental health or psychological distress. Analysing mood states through specific dimensions such as tension, depression, anger, fatigue, and vigour may provide a more precise understanding of students’ affective functioning and help identify modifiable factors associated with more favourable or unfavourable profiles.

This perspective is aligned with the need to develop more integrated, person-centred, and preventive health promotion strategies. Although the present study focuses on university students, its findings may be relevant for collaborative approaches involving university services, health education initiatives, PA professionals, psychological support services, and primary care. In this sense, the assessment of mood states alongside modifiable lifestyle behaviours may contribute to identifying student profile that could benefit from sleep health promotion, PA guidance, or psychosocial support.

The present study was conceptually grounded in the biopsychosocial model of health, which proposes that health-related outcomes emerge from the interaction of biological, psychological, behavioural, and social factors rather than from isolated determinants. Within this framework, sleep and physical activity represent modifiable behavioural dimensions, mood states reflect psychological functioning, and sociodemographic and academic characteristics constitute relevant contextual factors. This theoretical perspective therefore provides an appropriate basis for examining how lifestyle behaviours and contextual characteristics are associated with university students’ affective functioning.

Accordingly, the present study aimed to analyse mood-state profiles in university students and to examine their associations with PA, sleep quality, sleep duration, and sociodemographic and academic characteristics. Based on previous evidence, we hypothesised that poorer sleep quality and lower levels of PA would be associated with higher scores in negative mood-state dimensions (depression, fatigue, anger, and tension) and lower scores in vigour. We also hypothesised that more favourable sleep-related indicators would be associated with a more positive mood-state profile. Associations with sociodemographic and academic characteristics were examined as complementary analyses, given the more heterogeneous evidence available for these factors.

## 2. Materials and Methods

### 2.1. Study Design and Participants

This study used a cross-sectional, non-experimental, and analytical design. A quantitative approach with descriptive, comparative, and correlational components was adopted to analyse the relationship between PA, sleep quality, sleep duration, and mood states in university students.

Participants were recruited through non-probabilistic convenience sampling among students enrolled at a Spanish university. Accordingly, the sample was not intended to be representative of the national university student population. Initially, 1569 students participated in the study. The inclusion criteria were as follows: (a) being enrolled as a university student during the data collection period and (b) voluntarily agreeing to participate in the study by providing informed consent. The exclusion criteria were: (a) reporting medical conditions that could interfere with the study variables; (b) being under pharmacological treatment related to sleep disorders or psychological alterations; and (c) providing incomplete questionnaires or invalid responses.

After applying these criteria, 100 participants were excluded. The final sample consisted of 1469 university students, of whom 432 were men and 1037 were women. The mean age of the participants was 21.59 ± 3.48 years. An a priori sample-size calculation was performed using G*Power version 3.1.9.7. Assuming a significance level of α = 0.05, a statistical power of 0.80, and an expected small-to-moderate effect size of 0.15, the estimated minimum sample size required was 346 participants. The final sample of 1469 participants therefore largely exceeded the minimum required sample size, providing adequate statistical power for the planned comparative and correlational analyses.

### 2.2. Instruments

#### 2.2.1. PA

PA was assessed using the short version of the International PA Questionnaire (IPAQ-SF), proposed by Craig et al. [25]. This instrument consists of seven items and collects information on the frequency, expressed as days per week, and duration, expressed as minutes per day, of PA performed during the previous seven days.

The questionnaire allows PA to be estimated at three intensity levels: walking or light PA, assigned 3.3 METs; moderate PA, assigned 4.0 METs; and vigorous PA, assigned 8.0 METs. Participants were classified according to their weekly PA level as follows: low PA when they did not meet the criteria for the moderate or high categories; moderate PA when they accumulated at least 600 MET-min/week; and high PA when they accumulated at least 3000 MET-min/week. In addition, the questionnaire includes an item assessing daily sedentary time. In the present study, the IPAQ-SF showed adequate internal consistency, with a Cronbach’s alpha value of α = 0.720.

#### 2.2.2. Sleep Quality and Sleep Duration

Sleep quality and sleep duration were assessed using the Pittsburgh Sleep Quality Index (PSQI), developed by Buysse et al. and validated in the Spanish population by Royuela and Macías [26]. The instrument consists of 19 self-administered questions that assess seven sleep components: subjective sleep quality, sleep latency, sleep duration, sleep efficiency, sleep disturbances, use of sleep medication, and daytime dysfunction.

Each component is scored on a scale from 0 to 3, where 0 indicates no difficulty and 3 indicates severe sleep disturbance. The sum of the seven components provides a global score ranging from 0 to 21. A score above 5 is commonly interpreted as indicative of poor sleep quality. In the present sample, the PSQI showed excellent internal consistency, with a Cronbach’s alpha value of α = 0.912.

Sleep duration was additionally categorised into three groups: <7 h/night, 7–9 h/night, and >9 h/night. These cut-off points were selected on the basis of previous evidence and established sleep recommendations. Chaput et al., based on data from 14 countries, reported that sleeping approximately 7–8 h per night was associated with more favourable physical and mental health indicators, whereas sleeping less than 7 h was associated with poorer health outcomes [27]. Accordingly, 7 h was adopted as the lower threshold for adequate sleep duration. In addition, the National Sleep Foundation recommends 7–9 h of sleep per night for young adults to support optimal health [28]. Therefore, participants sleeping 7–9 h/night were classified as being within the recommended range, whereas those sleeping <7 h/night and >9 h/night were classified below and above the recommended range, respectively.

#### 2.2.3. Mood States

Mood states were assessed using the Profile of Mood States (POMS). The abbreviated version validated by Andrade et al. was used [6]. This reduced version includes 29 items answered on a five-point Likert scale ranging from 0, “not at all”, to 4, “extremely”.

The instrument provides scores for five dimensions: vigour, considered the only positive-valence dimension, and four negative-valence dimensions: tension, depression, anger, and fatigue. Conceptually, tension reflects feelings of nervousness, restlessness, and heightened activation; depression reflects sadness, discouragement, and negative affect; anger captures feelings of irritation and hostility; fatigue reflects tiredness and reduced perceived energy; and vigour represents energy, enthusiasm, and psychological activation.

The vigour dimension includes items 2, 7, 12, 17, 22, and 27; tension includes items 1, 8, 11, 18, 21, and 28; depression includes items 3, 6, 13, 16, 23, and 26; anger includes items 4, 9, 14, 19, 24, and 29; and fatigue includes items 5, 10, 15, 20, and 25. Scores for each subscale were calculated by summing the corresponding items. Internal consistency was assessed for each POMS subscale. Cronbach’s alpha coefficients were 0.898 for depression, 0.877 for fatigue, 0.921 for anger, 0.740 for tension, and 0.878 for vigour. The overall POMS scale showed excellent internal consistency (α = 0.918).

#### 2.2.4. Sociodemographic and Academic Variables

Sociodemographic and academic information was collected using an ad hoc questionnaire. This questionnaire included questions on sex, age, perceived economic level, level of studies, field of knowledge, and average academic grade.

### 2.3. Procedure

Data collection was conducted through an online platform between November 2025 and March 2026. Participants were recruited through non-probabilistic convenience sampling. The invitation to participate was disseminated through social networks and institutional university mailing lists.

Before participation, students received information about the aims of the study, the voluntary nature of their participation, the confidentiality of the data, and their right to withdraw from the study at any time. All participants provided informed consent before accessing the assessment instruments.

The estimated completion time for the questionnaire was approximately 10 min. To encourage participation, students were entered into a draw in which they could receive financial compensation exchangeable for technological and/or educational material.

The study was conducted in accordance with the principles of the Declaration of Helsinki and complied with the guidelines established by the Ethics Committee of the University of Granada. Ethical approval was granted under reference number 3678/CEIH/2023.

### 2.4. Statistical Analysis

First, descriptive analyses were performed for the sociodemographic, academic, and lifestyle variables of the participants. Categorical variables were expressed as frequencies and percentages, whereas quantitative variables were presented as means and standard deviations. The internal consistency of the instruments was assessed using Cronbach’s alpha coefficient.

The normality of quantitative variables was examined using the Kolmogorov–Smirnov test. Since the analysed variables did not follow a normal distribution, non-parametric statistical tests were used.

The Mann–Whitney U test was used to analyse differences in mood-state dimensions according to dichotomous variables, including sex, academic level, and sleep quality. For variables with more than two categories, including perceived economic level, field of knowledge, academic grade, PA level, and sleep-duration category, the Kruskal–Wallis H test was applied. When the Kruskal–Wallis test was statistically significant, Dunn–Bonferroni pairwise post hoc comparisons were performed to identify specific between-group differences while controlling for multiple comparisons.

Effect sizes were additionally calculated to complement statistical significance and facilitate interpretation of the magnitude of between-group differences. For Mann–Whitney U comparisons, effect size was expressed as r, whereas epsilon-squared (ε^2^) was calculated for Kruskal–Wallis tests.

Bivariate associations between age, mood-state dimensions, total PA expressed as MET-min/week, and sleep duration expressed as hours per night were examined using Spearman’s rank-order correlation coefficient (ρ). PA and sleep duration were therefore entered as continuous variables in the correlation analyses, rather than as the categorical variables used for group comparisons. Ninety-five percent confidence intervals (95% CIs) for Spearman’s correlation coefficients were estimated using Fisher’s z transformation.

The level of statistical significance was set at *p* < 0.05 for all analyses. Statistical analyses were performed using IBM SPSS Statistics, version 28.0.

## 3. Results

### 3.1. Sample Characteristics

The sociodemographic, academic, and lifestyle characteristics of the sample are shown in Table 1. The final sample consisted of 1469 university students, of whom 432 were men (29.4%) and 1037 were women (70.6%). The mean age of the participants was 21.59 ± 3.48 years.

Most participants reported a medium perceived economic level (83.8%) and were enrolled in undergraduate studies (86.4%). Regarding field of knowledge, the highest proportion of participants belonged to Education Sciences (50.4%), followed by PA and Sport Sciences (12.7%), Health Sciences (8.8%), Social and Legal Sciences (8.3%), Sciences (8.3%), Arts and Humanities (7.8%), and Engineering and Architecture (3.7%).

In relation to academic performance, most students reported an average academic grade equivalent to “Very good” (68.9%). Regarding PA, 8.7% of participants were classified as inactive or having a low level of PA, 46.4% presented a moderate level, and 44.9% presented a high level. The mean PA score was 3071.31 ± 2016.97 MET-min/week.

With respect to sleep-related variables, 81.4% of students showed poor sleep quality, whereas 18.6% showed good sleep quality. Regarding sleep duration, 31.2% of participants reported sleeping less than 7 h per night, 67.5% slept between 7 and 9 h, and 1.2% reported sleeping more than 9 h. Mean sleep duration was 6.96 ± 1.18 h per night.

### 3.2. Differences in Mood States According to Sociodemographic and Academic Variables

Differences in mood-state dimensions according to sociodemographic and academic variables are presented in Table 2. Regarding sex, statistically significant differences were observed for fatigue and vigour. Women reported higher fatigue scores than men (7.00 ± 4.76 vs. 5.78 ± 4.44; *p* < 0.001; r = 0.119), whereas men reported higher vigour scores than women (13.69 ± 5.61 vs. 11.28 ± 5.20; *p* < 0.001; r = 0.197). No statistically significant sex differences were observed for depression, anger, or tension.

Perceived economic level was significantly associated with depression (H = 21.72, *p* < 0.001, ε^2^ = 0.013), fatigue (H = 11.00, *p* = 0.004, ε^2^ = 0.006), and anger (H = 6.25, *p* = 0.044, ε^2^ = 0.003). Dunn–Bonferroni post hoc comparisons showed that students reporting a low perceived economic level had higher depression scores than those reporting medium (adjusted *p* < 0.001) or high economic levels (adjusted *p* < 0.001), and higher fatigue scores than those in the medium (adjusted *p* = 0.007) and high groups (adjusted *p* = 0.020). Anger scores were also higher in the low than in the high economic-level group (adjusted *p* = 0.042). No statistically significant differences were observed for tension or vigour. Effect sizes were small.

Regarding academic level, statistically significant differences were observed only for anger. Undergraduate students reported higher anger scores than postgraduate students (5.50 ± 5.25 vs. 3.95 ± 4.49; *p* < 0.001; r = 0.106). No statistically significant differences were observed for depression, fatigue, tension, or vigour.

Field of knowledge was significantly associated with fatigue (H = 33.09, *p* < 0.001, ε^2^ = 0.019) and vigour (H = 72.06, *p* < 0.001, ε^2^ = 0.045). Dunn–Bonferroni post hoc comparisons showed that students in Arts and Humanities had higher fatigue scores than students in PA and Sport Sciences (adjusted *p* < 0.001) and Education Sciences (adjusted *p* < 0.001), while students in Social and Legal Sciences also showed higher fatigue scores than those in PA and Sport Sciences (adjusted *p* = 0.020). For vigour, students in PA and Sport Sciences showed significantly higher scores than students from all other fields of knowledge (all adjusted *p* ≤ 0.005). No statistically significant differences according to field of knowledge were observed for depression, anger, or tension. Effect sizes were small.

Academic grade was significantly associated with depression (H = 22.74, *p* < 0.001, ε^2^ = 0.013), fatigue (H = 10.71, *p* = 0.013, ε^2^ = 0.005), anger (H = 14.02, *p* = 0.003, ε^2^ = 0.008), tension (H = 8.42, *p* = 0.038, ε^2^ = 0.004), and vigour (H = 27.99, *p* < 0.001, ε^2^ = 0.017). Dunn–Bonferroni post hoc comparisons showed significantly lower depression scores among students with Very good and Excellent grades compared with several lower-grade categories. Excellent-grade students also showed lower anger scores than the Pass, Good, and Very good groups, while tension differed significantly only between the Pass and Excellent groups. For vigour, students with Very good and Excellent grades showed significantly higher scores than those in the Good category. Although the omnibus test for fatigue was statistically significant, no individual pairwise comparison remained statistically significant after adjustment for multiple comparisons. Effect sizes were small across all academic-grade comparisons.

### 3.3. Mood States According to Physical Activity and Sleep Variables

Differences in mood-state dimensions according to PA and sleep variables are presented in Table 3. PA level was significantly associated with depression (H = 9.99, *p* = 0.007, ε^2^ = 0.005), fatigue (H = 14.49, *p* < 0.001, ε^2^ = 0.009), anger (H = 7.64, *p* = 0.022, ε^2^ = 0.004), tension (H = 7.34, *p* = 0.026, ε^2^ = 0.004), and vigour (H = 112.66, *p* < 0.001, ε^2^ = 0.075). Dunn–Bonferroni post hoc comparisons showed that the inactive/low-PA group had significantly higher depression scores than both the moderate (adjusted *p* = 0.022) and high-PA groups (adjusted *p* = 0.005), and higher fatigue scores than the moderate (adjusted *p* = 0.023) and high-PA groups (adjusted *p* < 0.001). Anger was significantly higher in the inactive/low-PA group than in the moderate-PA group (adjusted *p* = 0.026). For tension, a significant difference was observed between the moderate- and high-PA groups (adjusted *p* = 0.038). Finally, vigour was significantly higher in the high-PA group than in both the inactive/low and moderate groups (both adjusted *p* < 0.001). Effect sizes were generally small, with the largest effect observed for vigour.

Sleep quality was significantly associated with all mood-state dimensions. Students with poor sleep quality showed higher depression (5.25 ± 4.99 vs. 2.26 ± 3.01; *p* < 0.001; r = 0.259), fatigue (7.19 ± 4.73 vs. 4.26 ± 3.71; *p* < 0.001; r = 0.251), anger (5.74 ± 5.28 vs. 3.34 ± 4.18; *p* < 0.001; r = 0.194), and tension (9.29 ± 4.61 vs. 7.49 ± 4.14; *p* < 0.001; r = 0.151) scores than students with good sleep quality. Conversely, students with good sleep quality showed higher vigour scores (13.79 ± 5.39 vs. 11.59 ± 5.36; *p* < 0.001; r = 0.155).

Sleep duration was significantly associated with depression (*p* < 0.001, ε^2^ = 0.027), fatigue (*p* < 0.001, ε^2^ = 0.029), anger (*p* < 0.001, ε^2^ = 0.008), tension (*p* < 0.001, ε^2^ = 0.009), and vigour (*p* < 0.001, ε^2^ = 0.010). Dunn–Bonferroni post hoc comparisons showed that participants sleeping <7 h/night had significantly higher depression, fatigue, anger, and tension scores and lower vigour scores than those sleeping 7–9 h/night (all adjusted *p* < 0.001). No statistically significant pairwise differences involving the >9 h/night group were observed after adjustment for multiple comparisons. Effect sizes were small.

Overall, most statistically significant between-group differences were associated with small effect sizes, indicating that the magnitude of several differences was limited despite statistical significance in this large sample. Comparatively larger effects were observed for sleep-quality differences and for vigour according to PA level.

### 3.4. Correlations Between Age, Mood States, PA, and Sleep Duration

Bivariate correlations between age, mood-state dimensions, PA, and sleep duration are presented in Table 4. Spearman’s rank-order correlations showed several statistically significant associations, although their magnitude varied substantially across variables.

Age was negatively correlated with anger (ρ = −0.090, 95% CI [−0.140, −0.039], *p* < 0.001) and sleep duration (ρ = −0.154, 95% CI [−0.204, −0.104], *p* < 0.001). No statistically significant associations were observed between age and depression, fatigue, tension, vigour, or PA.

The negative mood-state dimensions were positively intercorrelated. Depression showed positive correlations with fatigue (ρ = 0.714, 95% CI [0.688, 0.738], *p* < 0.001), anger (ρ = 0.701, 95% CI [0.674, 0.726], *p* < 0.001), and tension (ρ = 0.576, 95% CI [0.541, 0.609], *p* < 0.001). Fatigue was also positively correlated with anger (ρ = 0.572, 95% CI [0.537, 0.605], *p* < 0.001) and tension (ρ = 0.514, 95% CI [0.475, 0.551], *p* < 0.001), while anger and tension showed a positive correlation (ρ = 0.657, 95% CI [0.627, 0.685], *p* < 0.001).

Vigour showed negative correlations with depression (ρ = −0.070, 95% CI [−0.121, −0.019]) and fatigue (ρ = −0.190, 95% CI [−0.239, −0.140]), and positive correlations with anger (ρ = 0.134, 95% CI [0.083, 0.184], *p* < 0.001) and tension (ρ = 0.324, 95% CI [0.277, 0.369], *p* < 0.001).

PA was negatively correlated with depression (ρ = −0.081, 95% CI [−0.132, −0.030], *p* = 0.002) and fatigue (ρ = −0.118, 95% CI [−0.168, −0.067], *p* < 0.001), and positively correlated with vigour (ρ = 0.287, 95% CI [0.239, 0.333], *p* < 0.001). No statistically significant correlations were observed between PA and anger (ρ = −0.032, 95% CI [−0.083, 0.019]) or tension (ρ = −0.034, 95% CI [−0.085, 0.017]).

Sleep duration was negatively correlated with depression (ρ = −0.192, 95% CI [−0.241, −0.142], *p* < 0.001), fatigue (ρ = −0.206, 95% CI [−0.254, −0.157], *p* < 0.001), anger (ρ = −0.113, 95% CI [−0.163, −0.062], *p* < 0.001), and tension (ρ = −0.120, 95% CI [−0.170, −0.069], *p* < 0.001), and positively correlated with vigour (ρ = 0.122, 95% CI [0.071, 0.172], *p* < 0.001). PA was positively correlated with sleep duration (ρ = 0.388, 95% CI [0.344, 0.431], *p* < 0.001).

## 4. Discussion

The present study aimed to analyse the profile of mood states in university students and to examine its relationship with PA, sleep quality, and sleep duration, while also considering sociodemographic and academic variables. Overall, the findings showed that less favourable sleep-related indicators and lower PA levels were associated with a less favourable mood-state profile. Poorer sleep quality and shorter sleep duration were associated with higher scores in negative mood-state dimensions, whereas vigour showed a more favourable pattern in relation to PA and sleep. These findings generally supported the initial hypotheses and highlight the relevance of considering both lifestyle behaviours and contextual characteristics when examining affective functioning in university students.

From a descriptive perspective, one of the most relevant findings was the high prevalence of poor sleep quality, observed in 81.4% of the sample. In addition, mean sleep duration was 6.96 ± 1.18 h/night, and 31.2% of participants reported sleeping less than 7 h per night. These findings indicate that sleep-related difficulties in this university population involved both qualitative and quantitative dimensions of sleep. This distinction is relevant because sleep health is a multidimensional construct that encompasses not only duration, but also continuity, efficiency, sleep latency, subjective satisfaction, and daytime functioning [29,30,31,32].

The high prevalence of poor sleep quality among university students is an alarming finding from a preventive perspective. Previous literature has indicated that sleep problems in university students may be associated with multiple factors, including high academic demands, workload, stress, frequent consumption of alcoholic and stimulant beverages, and certain night-time habits, such as screen use before bedtime [33]. These factors may interfere with the continuity and restorative perception of sleep, thereby affecting students’ daytime functioning. Likewise, poorer sleep quality has been associated with daytime sleepiness, lower academic performance, and greater difficulties in emotional regulation, reinforcing the need to address sleep as a central component of university well-being [34].

Regarding sleep quality, the results showed that students with poor sleep quality obtained higher scores in depression, fatigue, anger, and tension, whereas those who reported good sleep quality obtained higher scores in vigour. This finding suggests that sleep quality may constitute a relevant indicator of the affective profile of university students. Previous literature has shown that sleep plays a fundamental role in emotional regulation and cognitive functioning; therefore, poorer sleep quality may be related to greater psychological discomfort and a lower perceived capacity to cope with academic demands [35].

Previous research has proposed several mechanisms through which sleep disturbances and physical activity may be associated with affective functioning, including emotional-regulation processes and possible neuroendocrine, inflammatory, and circadian pathways [36]. However, the present study did not assess neurobiological or physiological mechanisms; therefore, the processes underlying the observed associations cannot be determined from these data. At the behavioural level, our findings indicate that poorer self-reported sleep quality was associated with a less favourable mood-state profile, characterised by higher depression, fatigue, anger, and tension and lower vigour.

In the university context, this relationship could intensify during periods of high academic pressure, such as examination periods, in which insufficient and poor-quality sleep has been associated with higher levels of stress, anxiety, and fatigue [37]. However, this relationship appears to be bidirectional, as negative mood states may also make it more difficult to initiate and maintain sleep. In this line, Olarte-Durand et al. reinforce the idea that anxiety acts as a factor that significantly increases the likelihood of experiencing poor sleep quality, generating a reciprocal interaction between emotional discomfort and non-restorative sleep [38]. The results for sleep duration complement those observed for sleep quality. Shorter sleep duration was significantly associated with higher depression, fatigue, anger, and tension, and with lower vigour. Similarly, categorical comparisons showed significant differences across sleep-duration groups in all five mood-state dimensions. Although these associations were generally small in magnitude, their consistent direction suggests that sleep quantity may also be relevant to students’ affective functioning. Taken together, the findings highlight the importance of considering sleep quality and sleep duration as complementary, rather than interchangeable, dimensions of sleep health.

Regarding PA levels, although most students reported moderate or high levels of practice, a smaller proportion of the sample was classified as inactive/low PA. This group showed the least favourable descriptive mood-state profile, with the highest scores in depression, fatigue, anger, and tension, whereas students with high PA showed the highest vigour scores. This pattern is broadly consistent with previous literature linking lower PA and greater sedentary behaviour with poorer psychological well-being in university populations [39,40,41]. From this perspective, PA may constitute a relevant behavioural correlate of affective functioning, as previous studies have associated greater PA with higher positive affect, better perceived well-being, and more adaptive coping strategies [42].

The correlational analyses provided additional nuance to these group comparisons. Higher total PA was associated with lower depression and fatigue, and with higher vigour. However, PA was not significantly correlated with anger or tension. These findings suggest that the association between PA and mood states may differ across affective dimensions and that statistically significant categorical group differences should not be interpreted as evidence of uniformly strong linear associations. Overall, the results are consistent with previous studies indicating that PA and psychological well-being are closely related in university students [43,44].

Similarly, some studies have suggested that physical inactivity may be related to psychological processes involved in emotional regulation, positive affect, and coping with academic stress [45]. In this sense, students with lower levels of PA may present less adaptive coping strategies, lower resilience, and a greater tendency to interpret academic difficulties negatively [46].

Regarding age, the correlational analyses showed significant negative associations only with anger and sleep duration. No statistically significant associations were observed between age and depression, fatigue, tension, vigour, or PA. The magnitude of the significant correlations was small, suggesting that age played a limited role in explaining differences in mood-state profiles within this relatively young university sample.

In the present study, the results according to sex showed that women obtained higher fatigue scores than men. This finding is consistent with recent studies that have reported higher levels of emotional exhaustion, perceived stress, and academic fatigue among female university students [47,48]. Cuevas et al. argue that these differences may be linked to emotional expression and stress management, given that in areas such as health and teaching, women face a higher risk due to the emotional and caregiving demands typical of these professions [49]. In this regard, recent studies have linked fatigue in university populations to academic stress, anxiety in demanding situations, and concern about completing tasks and projects [50,51].

Continuing with the differences observed according to sex, men showed higher scores in the vigour dimension than women, coinciding with previous studies in university populations [52]. From a sociocultural perspective, the differences observed in vigour could be partially related to gender socialisation patterns that influence the way men and women perceive and express energy, vitality, and disposition toward action [53]. However, this finding should be interpreted with caution, since vigour assessed through the POMS is an affective and subjective dimension. This interpretation should also consider the sex imbalance in the sample, with women representing 70.6% of participants and men 29.4%, which may have affected the robustness of sex-based comparisons. Moreover, the differences found could also be mediated by differential patterns of PA, as several studies indicate that male university students usually present higher levels of PA practice [54]. In this sense, greater involvement in physically active behaviours could contribute to a higher subjective perception of energy and vitality.

Perceived economic level was also associated with specific mood-state dimensions. Students reporting a low perceived economic level showed higher depression, fatigue, and anger scores than students in more favourable economic categories. These findings are consistent with previous literature identifying socioeconomic disadvantage as a relevant correlate of university students’ emotional well-being. In this sense, Montero et al. and Platania showed that lower family income is associated with greater anxious and depressive symptomatology, partly because financial uncertainty may constitute an additional source of stress [55,56]. Similarly, Rodrigues et al. identified economic difficulties as a factor associated with greater emotional distress. These findings suggest that perceived financial pressure may contribute to a less favourable affective profile among students with fewer economic resources [57].

Regarding academic level, statistically significant differences were observed only for anger, with undergraduate students showing higher scores than postgraduate students. Given that no significant differences were observed for depression, fatigue, tension, or vigour and that the magnitude of the observed difference was small, this result should be interpreted cautiously. Nevertheless, it may reflect specific demands associated with undergraduate study, including adaptation to university life, evaluative pressure, and academic or vocational uncertainty.

In relation to field of knowledge, significant differences were observed for fatigue and vigour. Students in Arts and Humanities showed higher fatigue scores than those in PA and Sport Sciences and Education Sciences, while students in Social and Legal Sciences also showed higher fatigue than those in PA and Sport Sciences. Previous research suggests that differences between academic disciplines may partly reflect variations in workload, task organisation, assessment demands, and the characteristics of specific degree programmes [58]. In contrast, vigour was significantly higher among students in PA and Sport Sciences than among students from all other fields of knowledge. This finding may be related to the more physically active profile typically associated with these degrees, since students enrolled in physical education and sport-related programmes tend to report greater habitual PA [59]. Greater engagement in active behaviours could therefore contribute to a higher subjective perception of energy and vitality [60], although the observed differences were small in magnitude.

Regarding academic grade, statistically significant differences were observed across all mood-state dimensions, although the magnitude of these differences was generally small and the pattern of significant pairwise comparisons varied depending on the specific dimension analysed. Students with higher academic grades tended to show a more favourable affective profile, particularly in relation to depression, anger, tension, and vigour. Specifically, lower depression scores were observed among students with Very good and Excellent grades compared with several lower-grade categories, whereas Excellent-grade students also showed lower anger scores than students in the Pass, Good, and Very good groups. For tension, the significant pairwise difference was restricted to the comparison between the Pass and Excellent categories. In the case of vigour, students with Very good and Excellent grades showed higher scores than those in the Good category. By contrast, although the omnibus test for fatigue was statistically significant, none of the individual pairwise comparisons remained significant after adjustment for multiple comparisons. These results suggest that academic performance is associated with specific aspects of students’ affective functioning, although the generally small effect sizes indicate that these differences should be interpreted cautiously rather than as evidence of a strong or uniform relationship across all mood dimensions.

Previous evidence supports the existence of a bidirectional relationship between psychological well-being and academic performance. On the one hand, negative affective states may interfere with cognitive and behavioural processes that are relevant for academic functioning, including concentration, motivation, study planning, persistence, and the ability to cope effectively with academic demands. On the other hand, academic difficulties, repeated experiences of failure, or lower-than-expected performance may themselves contribute to frustration, worry, perceived incompetence, and greater emotional discomfort [61,62]. In this sense, the association observed in the present study should not be interpreted as indicating a unidirectional effect of mood states on academic performance, or vice versa, particularly given the cross-sectional nature of the study.

Academic self-efficacy may constitute one of the psychological mechanisms contributing to this relationship. Students who perceive themselves as capable of successfully managing academic demands may be more likely to maintain motivation, persistence, and adaptive coping strategies when facing academic difficulties. Conversely, students with poorer academic results may experience a lower perception of competence, which could contribute to less favourable affective states. Previous studies have linked higher academic self-efficacy with better psychological adjustment, greater academic engagement, and more favourable academic outcomes [63]. Nevertheless, as academic self-efficacy was not directly assessed in the present study, this mechanism should be considered as a possible explanatory pathway rather than a conclusion derived from our data.

The results of this study support a multidimensional and integrated perspective on university students’ affective well-being, in which lifestyle behaviours and contextual characteristics are jointly relevant. Sleep emerged as a particularly important factor. Poor sleep quality was highly prevalent, affecting 81.4% of the sample, while mean sleep duration was 6.96 ± 1.18 h/night and 31.2% of participants reported sleeping less than 7 h/night. Both poorer sleep quality and shorter sleep duration were associated with a less favourable mood-state profile, although the magnitude of several associations was small. These findings support considering sleep quality and sleep duration as complementary dimensions of sleep health.

PA was also associated with mood-state profiles. Students with inactive/low PA generally showed a less favourable profile, whereas those with high PA showed greater vigour. At the correlational level, higher PA was associated with lower depression and fatigue and higher vigour, while no significant linear associations were observed with anger or tension. In addition, sex, perceived economic level, academic level, field of knowledge, and academic grade were associated with specific mood-state dimensions, although most between-group effect sizes were small.

All these findings reinforce the need for institutions to stop viewing health as solely an individual responsibility and to begin implementing comprehensive university strategies for well-being promotion that act upon the structural conditions of the campus. From the perspective of the Special Issue, these results also support the relevance of integrated approaches that connect PA, sleep health, affective well-being, psychosocial vulnerability, and preventive action. In this sense, university settings may represent strategic spaces for health education and early identification of unfavourable mood-state profiles, while collaboration with health professionals and primary care services could strengthen the continuity and sustainability of preventive and person-centred interventions.

## 5. Limitations and Future Research

Despite the relevance of the findings, this study is not without limitations, which should be taken into account when interpreting the results.

First, the study was based on a cross-sectional design. Since data were collected at a single time point, it is not possible to establish causal relationships between the variables analysed. Therefore, the associations observed between PA, sleep quality, sleep duration, and mood states should be interpreted as relational rather than causal.

Second, although the sample was large, it was obtained through non-probabilistic convenience sampling and was focused on university students from Granada. This geographical limitation may affect the external validity of the results, since environmental characteristics, climate, academic rhythms, and local university dynamics may differ from those of students from other regions, such as northern Spain. Consequently, the generalisation of these findings to the entire national university population should be made with caution.

Third, there was a significant imbalance in the sample according to sex, with a higher proportion of women (70.6%) than men (29.4%). This lack of balance may have influenced those results in which sex appeared as a differentiating variable, particularly in fatigue and vigour. Future studies should aim to recruit more balanced samples in order to analyse sex-based differences with greater precision.

Fourth, the sample also showed an unequal distribution according to field of knowledge. Most participants belonged to Education Sciences, whereas other areas, such as Engineering and Architecture, were underrepresented. This imbalance limits the possibility of generalising the results to all faculties and academic disciplines. Future research should include more balanced participation across fields of knowledge in order to better understand whether academic context influences mood-state profiles.

Fifth, the study relied on self-reported questionnaires and did not include objective measures. Although the instruments used showed adequate or excellent internal consistency, the results depend on participants’ personal and subjective perceptions, as well as on their recall capacity. This may lead to unintentional errors, social desirability bias, or inaccuracies in the estimation of PA and sleep. In addition, the absence of objective measures prevents comparison between students’ self-reported information and objective indicators of PA or sleep.

Based on these limitations, several recommendations for future research can be proposed. First, longitudinal studies should be designed to follow students across different periods of the academic year, for example, comparing examination periods with ordinary teaching periods. This type of design would allow for a more consistent analysis of possible causal relationships and would make it possible to observe the long-term effect of lifestyle habits on affective profiles.

Second, future studies should broaden and diversify the sample by including universities from different geographical and cultural contexts. In addition, more specific recruitment strategies should be applied to favour a balanced representation according to sex and field of knowledge. This would increase the presence of currently underrepresented areas, such as Engineering and Architecture, and would facilitate greater generalisation of the findings to the broader university context.

Third, future research should consider incorporating objective measures of PA and sleep, such as accelerometers, actigraphy, or wearable devices. These tools would make it possible to complement self-reported data and obtain a more precise assessment of students’ lifestyle behaviours. Likewise, future studies could include additional psychosocial variables, such as academic stress, perceived social support, resilience, self-efficacy, or time management, in order to better understand the mechanisms linking lifestyle habits and mood states.

## 6. Practical Implications

The results obtained in this study allow several practical implications to be derived for the promotion of well-being and healthy lifestyle habits in university students.

First, the high prevalence of poor sleep quality observed in this study highlights the need to implement sleep hygiene programmes in the university context. Specifically, 81.4% of students presented poor sleep quality regardless of the number of hours slept. Therefore, interventions should not focus exclusively on sleep duration, but also on the continuity, efficiency, regularity, and restorative perception of sleep. Programmes aimed at improving sleep routines, reducing night-time screen exposure, promoting relaxation strategies, and managing academic stress may contribute to improving students’ quality of life and affective profiles.

Second, the negative association observed between PA and depression reinforces the role of PA as a relevant modulator of affective well-being. In this regard, universities should promote free and accessible PA programmes within the campus in order to reduce the percentage of inactive students detected in this study. These initiatives should be inclusive, adapted to different levels of physical condition, and integrated into students’ daily routines. However, they should also be complemented with guidance on time management and personal organisation, in order to prevent sport practice from being perceived as an additional burden that may increase tension among students facing high academic demands.

Third, the findings point to the need to detect and support vulnerable profiles. Students with a low perceived economic level showed a less favourable mood-state profile, with higher scores in depression, fatigue, and anger. Therefore, universities should strengthen early detection systems and provide psychoeducational, academic, and economic support to reduce the stress derived from financial uncertainty. These actions may be especially relevant for students who combine academic demands with economic difficulties or limited access to support resources.

Fourth, comprehensive university well-being programmes should be implemented. Although the main variable analysed in this study was mood states, the findings show that affective functioning is closely related to sleep, PA, academic performance, and psychosocial factors. Therefore, student well-being should not be addressed in isolation, but as a key component of academic success. Given that students with better academic grades showed higher vigour and lower negative mood-state scores, faculties should integrate the promotion of healthy habits into their institutional culture. This may include flexible academic planning during periods of high pressure, the creation of spaces for emotional disconnection and physical activation, and the coordination of academic, psychological, and health promotion services.

Finally, the results support the need for an institutional approach to prevention. Universities should not place responsibility for well-being solely on students but should take an active role in creating favourable conditions within the educational environment. This involves reviewing academic workloads and timetables to reduce excessive demands that may increase tension and deteriorate sleep quality. Likewise, universities should guarantee free and equitable access to PA programmes and provide structural support for students who are more vulnerable due to their economic situation or field of study. Affective well-being should be managed as a central component of academic success and not as an optional or exclusively individual matter.

From an integrated health promotion perspective, these implications are also relevant beyond the university context. Collaboration between university services, primary care professionals, PA specialists, psychologists, nurses, and health educators could help to identify students with unfavourable mood-state profiles and provide coordinated guidance on sleep, PA, and psychosocial support. This collaborative approach would be consistent with person-centred and preventive models that integrate physical, affective, and psychosocial dimensions of health.

## 7. Conclusions

This study highlights the relevance of both lifestyle behaviours and contextual characteristics in university students’ mood-state profiles. Poor sleep quality was highly prevalent, and almost one-third of participants reported sleeping less than 7 h/night. Poorer sleep quality and shorter sleep duration were consistently associated with a less favourable mood-state profile, although the magnitude of several associations was small. These findings support considering sleep quality and sleep duration as complementary dimensions of sleep health.

PA was also associated with affective functioning. Students with inactive/low PA generally showed a less favourable mood-state profile, whereas students with high PA showed greater vigour. Correlational analyses further indicated that higher PA was associated with lower depression and fatigue and higher vigour, while no significant associations were observed with anger or tension.

Sociodemographic and academic characteristics were associated with specific mood-state dimensions. In particular, differences were observed according to sex, perceived economic level, academic level, field of knowledge, and academic grade; however, most between-group effect sizes were small. These results support a cautious interpretation of statistically significant differences and highlight the multifactorial nature of affective functioning in university students.

Overall, the results highlight the need to rethink the role of universities as settings for integrated health promotion. Beyond academic evaluation, universities should become environments that promote students’ physical, affective, and psychosocial well-being. In this sense, coordinated strategies focused on sleep quality, healthy and accessible PA, early detection of vulnerable profiles, and institutional support may contribute to more sustainable, preventive, and person-centred approaches to university well-being. These findings may also inform collaborative actions between university services, health education initiatives, and primary care professionals aimed at improving the affective profiles and overall well-being of young adults.

## Figures and Tables

**Table 1:** Sociodemographic, academic, and lifestyle characteristics of the sample.

Characteristics	Category	Frequency (n)	Percentage (%)
Sex	Men	432	29.4
	Women	1037	70.6
Perceived economic level	Low	161	11.0
	Medium	1232	83.8
	High	76	5.2
Academic level	Undergraduate	1269	86.4
	Postgraduate	200	13.6
Field of knowledge	PA and Sport Sciences	187	12.7
	Education Sciences	741	50.4
	Social and Legal Sciences	122	8.3
	Engineering and Architecture	54	3.7
	Arts and Humanities	114	7.8
	Health Sciences	129	8.8
	Sciences	122	8.3
Academic grade	Pass	29	2.0
	Good	221	15.0
	Very good	1012	68.9
	Excellent	207	14.1
PA level	Inactive/low	128	8.7
	Moderate	681	46.4
	High	660	44.9
Sleep quality	Poor sleep quality	1197	81.4
	Good sleep quality	272	18.6
Sleep duration	Less than 7 h	459	31.2
	Between 7 and 9 h	992	67.5
	More than 9 h	18	1.2
		M ± SD	
Age		21.59 ± 3.48	
PA (MET-min/week)		3071.31 ± 2016.97	
Sleep duration (hours)		6.96 ± 1.18	

**Table 2:** Mood-state dimensions according to sociodemographic and academic variables.

Variables		Depression	*p*	Fatigue	*p*	Anger	*p*	Tension	*p*	Vigour	*p*
Sex	Men	4.51 ± 4.61	0.565	5.78 ± 4.44	<0.001	4.95 ± 5.01	0.093	9.09 ± 4.50	0.533	13.69 ± 5.61	<0.001
	Women	4.77 ± 4.92		7.00 ± 4.76		5.43 ± 5.23		8.91 ± 4.62		11.28 ± 5.20	
Perceived economic level	Low	6.68 ± 6.02	<0.001	7.95 ± 5.34	0.004	6.25 ± 5.94	0.044	9.78 ± 5.03	0.078	11.18 ± 5.89	0.120
	Medium	4.49 ± 4.61		6.51 ± 4.57		5.23 ± 5.09		8.88 ± 4.52		12.07 ± 5.37	
	High	3.80 ± 4.46		6.10 ± 4.99		4.19 ± 4.45		7.51 ± 4.50		13.39 ± 5.32	
Academic level	Undergraduate	4.71 ± 4.85	0.958	6.68 ± 4.65	0.257	5.50 ± 5.25	<0.001	9.02 ± 4.64	0.252	12.02 ± 5.36	0.730
	Postgraduate	4.60 ± 4.70		6.43 ± 5.01		3.95 ± 4.49		8.57 ± 4.17		11.85 ± 5.91	
Field of knowledge	PA and Sport Sciences	3.91 ± 4.19	0.100	5.68 ± 3.970	<0.001	5.05 ± 4.94	0.149	8.93 ± 4.65	0.725	14.09 ± 5.14	<0.001
	Education Sciences	4.51 ± 4.70		6.21 ± 4.37		5.63 ± 5.30		9.00 ± 4.60		12.45 ± 5.19	
	Social and Legal Sciences	5.33 ± 5.33		7.57 ± 4.94		5.16 ± 5.25		9.00 ± 4.52		10.96 ± 5.65	
	Engineering and Architecture	5.06 ± 5.57		7.35 ± 4.73		4.13 ± 4.38		8.02 ± 4.28		9.72 ± 5.01	
	Arts and Humanities	5.81 ± 5.72		8.71 ± 5.60		5.26 ± 5.57		8.86 ± 4.77		9.73 ± 5.75	
	Health Sciences	4.55 ± 4.71		7.04 ± 4.98		4.77 ± 4.83		9.36 ± 4.41		11.22 ± 5.75	
	Sciences	5.30 ± 4.69		7.21 ± 5.38		4.82 ± 4.92		8.80 ± 4.56		11.00 ± 5.10	
Academic grade	Pass	7.10 ± 4.72	<0.001	8.52 ± 4.80	0.013	7.17 ± 5.40	0.003	10.90 ± 4.64	0.038	10.21 ± 5.23	<0.001
	Good	5.62 ± 5.17		7.23 ± 4.71		5.51 ± 5.20		9.04 ± 4.53		11.52 ± 6.09	
	Very good	4.53 ± 4.73		6.34 ± 4.49		5.38 ± 5.20		8.75 ± 4.66		12.30 ± 5.32	
	Excellent	4.14 ± 4.77		6.25 ± 4.72		4.33 ± 4.85		8.50 ± 4.67		12.45 ± 5.75	

Note. Data are presented as mean ± standard deviation. Differences between two groups were analysed using the Mann–Whitney U test, whereas differences among more than two groups were analysed using the Kruskal–Wallis H test. Statistical significance was set at *p* < 0.05.

**Table 3:** Mood-state dimensions according to PA and sleep variables.

Variables		Depression	*p*	Fatigue	*p*	Anger	*p*	Tension	*p*	Vigour	*p*
PA level	Inactive/low	6.05 ± 5.40	0.007	8.00 ± 5.04	<0.001	6.35 ± 5.67	0.022	9.41 ± 4.90	0.026	10.15 ± 4.93	<0.001
	Moderate	4.69 ± 4.83		6.78 ± 4.77		5.01 ± 5.12		8.61 ± 4.57		10.73 ± 5.32	
	High	4.43 ± 4.68		6.24 ± 4.51		5.37 ± 5.11		9.40 ± 4.90		13.65 ± 5.17	
Sleep quality	Poor	5.25 ± 4.99	<0.001	7.19 ± 4.73	<0.001	5.74 ± 5.28	<0.001	9.29 ± 4.61	<0.001	11.59 ± 5.36	<0.001
	Good	2.26 ± 3.01		4.26 ± 3.71		3.34 ± 4.18		7.49 ± 4.14		13.79 ± 5.39	
Sleep duration	<7 h	5.87 ± 5.22	<0.001	7.90 ± 4.99	<0.001	5.99 ± 5.38	<0.001	9.61 ± 4.59	<0.001	11.15 ± 5.41	<0.001
	7–9 h	4.14 ± 4.54		6.06 ± 4.46		4.97 ± 5.05		8.65 ± 4.54		12.39 ± 5.40	
	>9 h	5.11 ± 4.84		7.06 ± 4.11		5.22 ± 5.34		9.44 ± 5.23		11.83 ± 5.31	

Note. Effect-size estimates and adjusted pairwise post hoc comparisons are reported in the accompanying text.

**Table 4:** Bivariate correlations between age, mood-state dimensions, PA, and sleep duration.

Variables	Age	Depression	Fatigue	Anger	Tension	Vigour	PA
Depression	0.002						
Fatigue	−0.018	0.714 ***					
Anger	−0.090 ***	0.701 ***	0.572 ***				
Tension	−0.020	0.576 ***	0.514 ***	0.657 ***			
Vigour	−0.030	−0.070 **	−0.190 ***	0.134 ***	0.324 ***		
PA	−0.040	−0.081 **	−0.118 ***	−0.032	−0.034	0.287 ***	
Sleep duration	−0.154 ***	−0.192 ***	−0.206 ***	−0.113 ***	−0.120 ***	0.122 ***	0.388 *

Note. Correlations were analysed using Spearman’s rank-order correlation coefficient (ρ). Ninety-five percent confidence intervals are reported in the Section 3. * *p* < 0.05; ** *p* < 0.01; *** *p* < 0.001.

## Data Availability

The data supporting the findings of this study are available from the corresponding author upon reasonable request. The data are not publicly available due to ethical and privacy restrictions related to the protection of participants’ personal information.

## Abbreviations

The following abbreviations are used in this manuscript:

IPAQ-SF	International Physical Activity Questionnaire-Short Form
PA	Physical Activity
POMS	Profile of Mood States
PSQI	Pittsburgh Sleep Quality Index
